# Survey data on income, food security, and dietary behavior among women and children from households of differing socio-economic status in urban and peri-urban areas of Nairobi, Kenya

**DOI:** 10.1016/j.dib.2020.106542

**Published:** 2020-11-19

**Authors:** Fridah N Nyakundi, Mercy Mutua, Mercy G Lung' aho, Christine K Chege, John Ndung' u, Rhoda Nungo, David Karanja

**Affiliations:** a International Centre for Tropical Agriculture (CIAT), P.O. Box 823-00621, Nairobi, Kenya; b Kenya Agricultural and Livestock Research Organization (KALRO), P.O. Box 57811-00200, Nairobi, Kenya

**Keywords:** Wealth status, Household food insecurity, Women, Children, Dietary diversity

## Abstract

This article describes data collected to analyze consumer behaviors in vulnerable populations by examining key access constraints to nutritious foods among households of differing socio-economic status in urban and peri‑urban areas of Nairobi, Kenya. The key variables studied include wealth status, food security, and dietary behavior indicators at individual and household level. Household food insecurity access scale (HFIAS), livelihood coping strategies (LCS), food expenditure share (FES), food consumption score (FCS), household dietary diversity score (HDDS), minimum dietary diversity-women(MDD-W), and child dietary diversity score (CDDS) indicators were used to measure food security. Household assets were used to develop an asset-based wealth index that grouped the study sample population into five wealth quantiles, while income levels were used to estimate FES. The hypothesis that guided the cross-sectional survey conducted to generate these data is that vulnerability to food insecurity and poverty are important drivers of food choice that influence household and individual dietary behavior. Data from this study was thus used to assess direction and strength of association between; household food insecurity, wealth status, women, children, and household dietary behavior in both urban and peri‑urban populations sampled.

**Specifications Table**

**Table:** 

Subject	Agricultural and Biological Sciences, Food Security and Nutrition
Specific subject area	Urban and peri‑urban consumer household food security and nutrition data
Type of data	Tables Chart Graphs Figures
How data were acquired	Survey questionnaire using Open Data Kit (ODK) - https://dataverse.harvard.edu/dataset.xhtml?persistentId=doi:10.7910/DVN/3JZKBO
Data format	Cleaned raw data Analyzed ODDS ratios tables accompanying this paper
Parameters for data collection	Households with a child aged between 6–59 months were randomly sampled from the study locations in Kiambu, Nairobi, and Machakos counties of Kenya.
Description of data collection	Data was collected using a face-to-face interview technique via a programmed questionnaire in the Open Data Kit (ODK).
Data source location	Institution: International Center for Tropical Agriculture (CIAT) City/Town/Region: Nairobi, Machakos, Kiambu Country: Kenya
Data accessibility	1. Survey data accompanying this article can be accessed via the URL link below: Repository name: Havard Dataverse Data identification number: 10.7910/DVN/3JZKBO Direct URL to data: https://dataverse.harvard.edu/dataset.xhtml?persistentId=doi:10.7910/DVN/3JZKBO 2. Analyzed data of individual food item consumption for women and children within the interviewed households is provided in this article.

## Value of the Data

• These data fill a significant knowledge gap and provide an overview of food security and a nutrition profile of low- and medium-income households in Nairobi's metropolitan urban and peri‑urban areas.
• This data can be used by local communities in which the survey was administered to address food security and nutrition challenges through community-led initiatives targeting the entire population and vulnerable groups such as children under five and pregnant women.
• Findings from these data can guide policymakers, advocacy teams, and implementers of food security interventions in the development of complementary and corrective policies and nutrition programming for vulnerable low- and medium-income households in Kenya's urban and peri‑urban areas.
• Program implementers can use these data to guide the development of appropriate interventions or to justify targeting for interventions for vulnerable households. The private sector, particularly the food processing sector, can use this information to develop affordable, innovative food products for low-income consumers.

## 1 Data Description

The data was collected in the context of a Cultivate Africa's Future (CultiAF) project on pre-cooked beans, led by the Kenya Agricultural and Livestock Research Organization (KALRO) and within/under the CGIAR Center Research Program (CRP) on Agriculture for Nutrition and Health (A4NH) in November 2015. A cross-sectional household survey was administered to collect nutrition, income, and demographic indicators used to assess access constraints to nutritious foods among urban and peri‑urban consumers. Urban consumers were sampled from three sub-counties of Nairobi County: Kibera, Dandora, and Mukuru Kwa Njenga. Peri-urban consumers were sampled from Athi River sub-county in Machakos County and Juja sub-County in Kiambu County. Data was collected using Open Data Kit (ODK). Sampling was undertaken among households with at least one child aged 6–59 months. In each household sampled, the target child, whose nutrition and anthropometric information was collected was indexed to track information collected. The primary caregiver of the index child, who was in most cases the child's mother, was purposively selected as the survey questionnaire respondent. The questionnaire used to collect the data has been edited and added to the Dataverse repository containing the data. A data dictionary which contains and describes all the 1147 data variables in the data is available Dataverse repository as Microsoft excel file adjacent to the dataset and questionnaire.

**Fig. 1:**
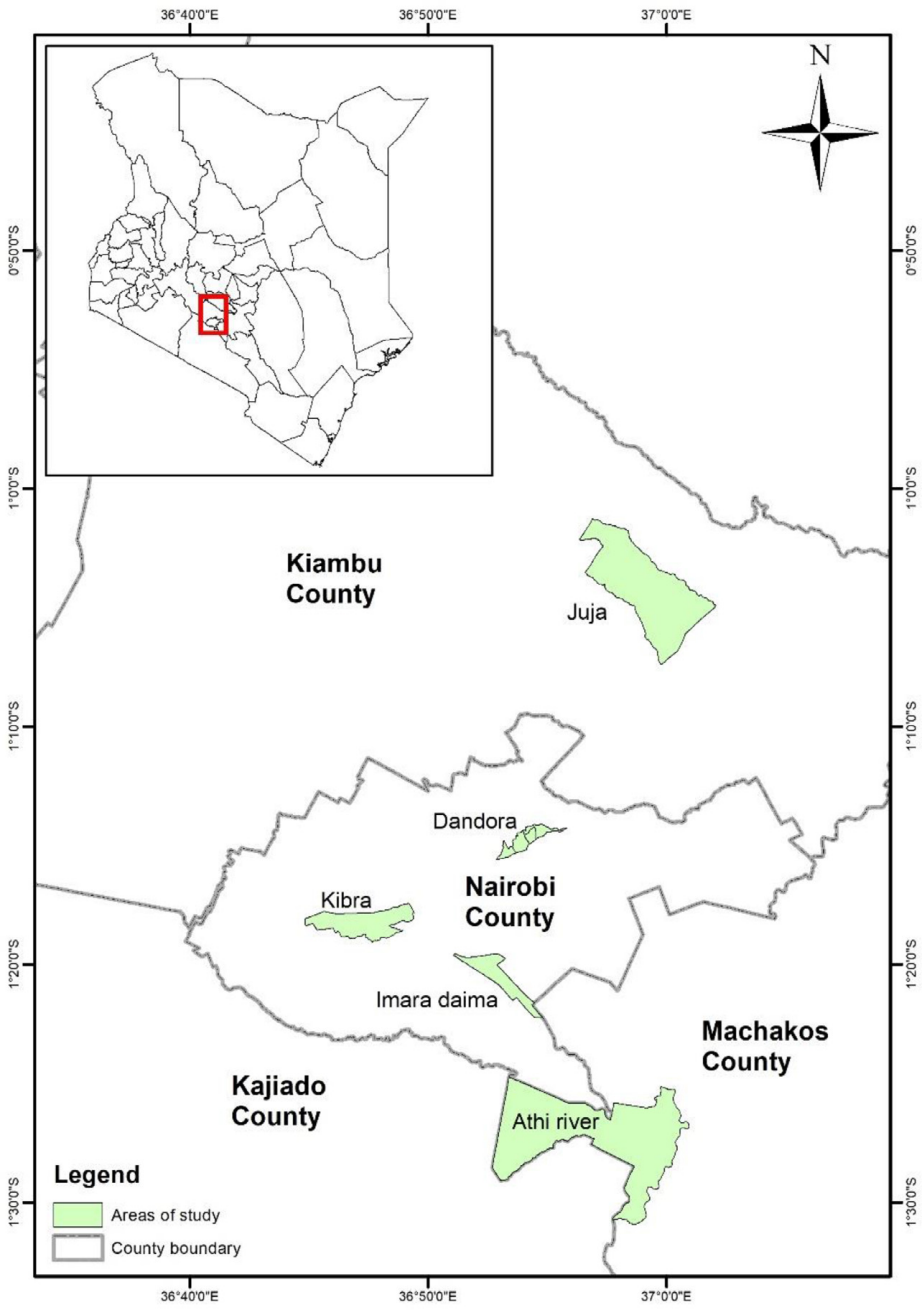
Map of Nairobi, Kiambu, and Machakos counties with study sites.

**Fig. 2:**
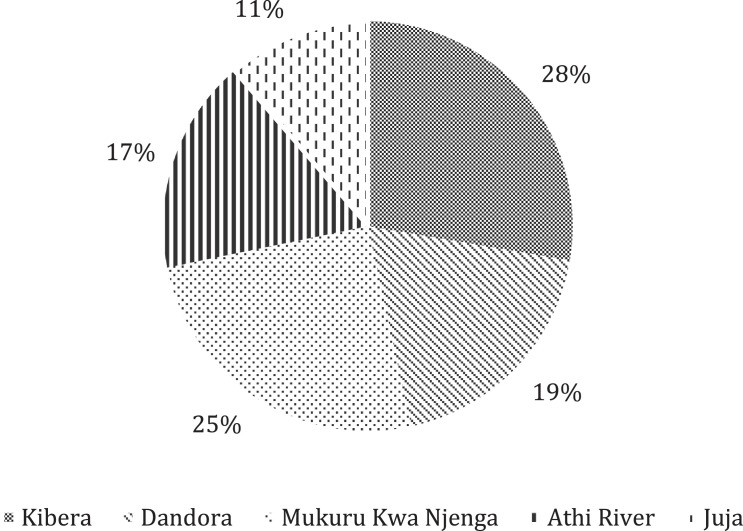
Proportion of respondents per location.

**Fig. 3:**
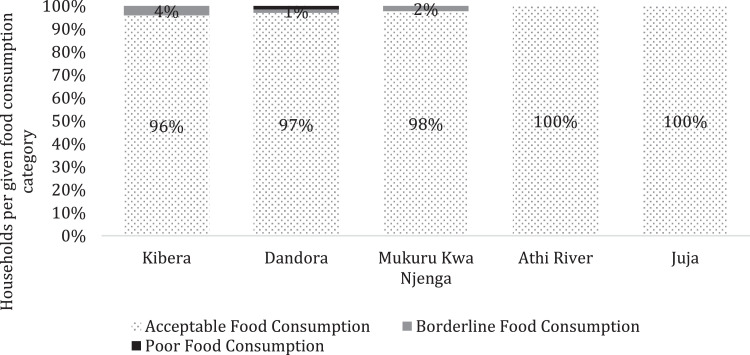
Summary of household food consumption score category per location.

**Fig. 4:**
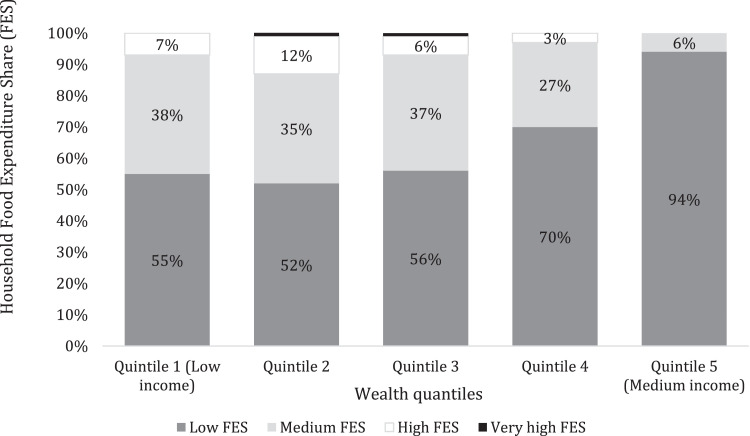
Summary of Food Expenditure Share (FES) by wealth quantile.

Fig. 1 presents the geographical study sites where data was collected, while Fig. 2 shows the respondents' distribution among the study sites. Fig. 3 presents the Food Consumption Score (FCS) that shows the diet diversity and frequency of food consumption characteristics, by showing the proportion of households with their corresponding FCS per location. Fig. 4 presents the household Food Expenditure Share (FES) used to measure household food security characteristics by acting as an income proxy. Table 1 presents the minimum women's diet diversity (MDD-W) characteristics by showing the proportion of women from each age group that consumed food from each of the ten food groups.

**Table 1:** A summary of the proportion of women that consumed food items from each food group per age-group.

Food Group	Women aged 15–25 years	Women aged 26–35 years	Women aged 36–49 years
Starch	100%	100%	100%
Other Vegetables	98%	98%	100%
DGLV	97%	95%	88%
Pulses	85%	88%	96%
Dairy	85%	85%	80%
Flesh	76%	78%	72%
Other Fruits	74%	77%	64%
Vitamin A-rich fruits and vegetables	70%	68%	60%
Eggs	63%	66%	40%
Nuts & seeds	28%	20%	12%

## 2 Experimental Design, Materials and Methods

### 2.1 Survey site and population

The study sites were purposely selected based on the Living Standards Measurement Study (LSMS) to include the following income classifications: low income (Kibera), upper-low income (Dandora and Athi River), and medium income (Mukuru Kwa Njenga, Juja, and Athi River). Population data from the Kenya National Bureau of Statistics (KNBS) census was used to determine the number and location of households sampled. A random sampling technique was employed to select sample households from a population of 327,745 households. A total of 354 households were sampled: Kibera (*N* = 98), Dandora (*N* = 67), Athi River(*N* = 60), Mukuru Kwa Njenga (*N* = 89), and Juja (*N* = 40). The sample size was calculated to achieve 80% power at α = 0.05 to ensure the study would detect the effect of either food insecurity or wealth inequality on poor dietary diversity at both the household and individual levels. Oral consent was obtained from the respondents before conducting the interviews. The survey targeted household representatives with adequate information on household food consumption, food intake by the index child (6–59 months), and index woman (biological mother or caregiver of the index child) as the study respondent.

### 2.2 Questionnaire modules

The questionnaire modules were designed to collect data to ascertain whether these three study hypotheses were true, for both urban and peri‑urban populations:

1. Households that are asset- or income-poor are food insecure.
2. Households that are income- or asset-poor have poor dietary diversity for the household, women, and children.
3. Households that are food insecure have poor dietary diversity for the household, women, and children.

The principal study variables collected included: household identification, household roster and demographics, dwelling characteristics, market access, household food consumption, household non-food expenditure, infant dietary diversity, women's dietary diversity, household hunger scale, hunger coping strategies, and household income sources.

Study questions that informed responses to the direction and association of the variables were:

1. Is it that wealth status affects food security, which then affects dietary behavior?
2. Or does food security, independent of wealth status, affect dietary behavior?
3. Is the pattern of strength and direction of association similar for both urban and peri‑urban populations?

### 2.3 Data collection and survey implementation

The questionnaire used for data collection was designed and then coded in Open Data Kit (ODK) to facilitate mobile data collection. The ODK form was hosted on a SurveyCTO cloud server. Enumerators used android tablets to collect data and transmitted it to the server on a daily basis. A data manager monitored the data received in the server and ran data quality checks for inconsistencies, patterns, and outliers, providing feedback to the field teams to improve performance.

Data were collected by trained enumerators associated with KALRO. All enumerators had a university bachelor's or higher-level degree. All spoke English and Kiswahili languages fluently and were experienced in data collection in urban and peri‑urban areas. Before data collection commenced, the enumerators received a four-day mandatory training on the questionnaire and Computer Assisted Personal Interviews (CAPI) enumeration skills using tablets.

The survey intentionally targeted one primary caregiver of the index child in each household, usually the mother, so as to improve the accuracy and detail of child dietary diversity scores (CDDS) and MDD-W parameters. As a result, 94% of the study respondents were women.

Data was collected using a face-to-face interview technique. The questionnaire was structured to generate both qualitative and quantitative data using a combination of open-ended and closed-ended questions.

### 2.4 Household characteristics

This module was used to collect data on household location, roster, demographics, and dwelling characteristics. Data on assets and dwelling characteristics were used to generate the household asset-based wealth index.

### 2.5 Household income sources

The household income module asked about the income sources from various livelihoods and the actual earnings in local currency for a recall period of twelve months.

### 2.6 Household non-food expenditure

The household non-food expenditure module split household non-food expenditures into a period of thirty days for ten common expenditures and for a period of six months for other expenses. Thirty-day recall items for recurring expenses that include rent, water, electricity, fuel, satellite, transport, garbage collection, household items, and addictive items such as alcohol and tobacco were recorded. Six-month recall expense items included medical fees, education fees, the debt amount, savings amount, house construction and house repairs, clothing, social events or celebration, and agricultural inputs. Estimates of all these household non-food expenditures and activities were recorded in local currency.

### 2.7 Household food consumption

The food consumption module sought to gather the household's current status of quality and quantity of food consumption seven days prior to the interview [1]. The food consumption module contained a list of sixteen food groups: (1) Cereals and grains, (2) Roots and tubers, (3) Legumes/nuts, (4) Orange vegetables (vegetables rich in Vitamin A), (5) Green leafy vegetables, (6) Other vegetables, (7) Orange fruits (fruits rich in Vitamin A), (8) Other fruits, (9) meat, (10) Liver, kidney, heart and other organ meats, (11) Fish/shellfish, (12) eggs, (13) Milk and other dairy products, (14) Oil / fat / butter, (15) Sugar/sweets, and (16) Condiments/spices. For each food group, the frequency of intake in the last seven days, the quantity of food consumed by the household, source of food consumed (purchased, non-purchased, or both), the estimated total cost of the food (cash, credit, or value of both for purchased and non-purchased food), and primary source of non-purchased food was indicated. An aggregate of food consumption frequency and diversity was used to calculate the individual FCS as per the World Food Programme (WFP) (2009) [2] guidelines, and Leroy et al. [1] as shown in Fig. 3.

The HFIAS data was collected using Coates’ et al. [3] nine questions, commonly referred to as items, using a 30-day recall period. The HFIAS indicator was constructed to measure the occurrence and frequency of the food insecurity dimension. While the individual items measured the food insecurity occurrence, categorical items were used to measure the frequency at which each item occurred. The HFIAS indicator was then calculated, and the score ranging from a minimum of zero (food-secure households), and a maximum score of 27 (food-insecure households) was allocated to each household, as shown in the data.

Using Smith and Subandoro's [4] measurement method, a combination of household food expenditure and non-food expenditure data was used to calculate FES. FES was used as a food security indicator and an income proxy for the households, as shown in Fig. 4. FES was used as an income proxy because findings from WFP (2009) [2] indicate that poor households had a higher share of total expenditures going towards food compared to wealthy households. This is especially true for households that depend mainly on purchased food instead of own production, which is the case in Nairobi metropolitan area, where this study was carried out. FES data was collected using a 30-day recall period.

The household food consumption module was designed to improve understanding of households' intake of key nutrient-rich foods. Aggregated data for food items from the 16 food groups, was used to construct HDDS to measure dietary behavior and food access, as proposed by Leroy et al. [1].

Finally, questionnaire responses from a series of nineteen questions were used to calculate the LCS indicator that measures the livelihood stress and assets depletion over a 30-day period before the survey as per WFP guidelines [2]. Respondents were classified into three broad categories depending on the food insecurity faced at household level. The LCS categories used in allocation include: *stress, crisis*, and *emergency coping.*

### 2.8 Child dietary diversity score(CDDS)

This module asked questions about food and drinks offered to the index child within a household to measure a child's diet diversity using CDDS as per the WFP and WHO guidelines [5,6]. The infant dietary diversity module also asked if the child had received vitamin drops, oral rehydration solution, or had drunk anything from a bottle with a teat. The module used a frequency of food and water intake over the 24 h prior to the study and whether the intake reported was usual or unusual [5,6].

The module contained a list of nine liquid foods given to the child: (1) Breast milk, (2) Plain water, (3) Infant formula, (4) Animal milk, (5) Juice, (6) Clear broth, (7) Yogurt, (8) Thin porridge, and (9) Any other liquid.

The module also contained a list of 16 food groups: (1) Cereals and grains, (2) Roots and tubers, (3) Legumes/nuts, (4) Orange vegetables [vegetables rich in Vitamin A], (5) Green leafy vegetables, (6) Other vegetables, (7) Orange fruits [fruits rich in Vitamin A], (8) Other fruits, (9) Meat, (10) Liver, kidney, heart and other organ meats, (11) Fish/shellfish, (12) Eggs, (13) Milk and other dairy products, (14) Oil / fat / butter, (15) Sugar/sweets, and (16) Foods made from red palm oil or red palm nut. Details about the children's dietary intakes were collected from their mothers or primary caregivers.

### 2.9 Women's dietary diversity

The Minimum Dietary Diversity – Women (MDD-W) indicator was used in this study as a proxy indicator to assess the micronutrient adequacy of diets consumed by women of reproductive age [7]. The dichotomous indicator had ten food groups: (1) All starchy staples, (2) Beans and peas, (3) Nuts and seeds, (4) Dairy, (5) Flesh foods, (6) Eggs, (7) Vitamin A-rich vegetables, (8) Vitamin A-rich dark green vegetables, (9) Other vegetables, (10) Other fruits.

Women were categorized into three age groups, 15 – 25 years, 26 – 35 years, and 36 – 49 years to check for the relationship between age and diet diversity.

### 2.10 Household hunger scale

The household hunger scale module used a set of eight questions to determine the occurrence of increasingly severe experiences of food shortage [8]. Four key module domains were assessed: worry about food access in the short term, uncertainty about food access in the long term, inadequate food quality, and insufficient food quantity. The recall period was 30 days. The module asked if the household had experienced any of the four domains, how often it occurred in the last 30 days, why the experience occurred, and who was affected – adults, children under 24 months, or both.

### 2.11 Livelihood coping strategies

The module used a set of 19 questions to determine how vulnerable households responded to food insecurity and what measures they took to mitigate the problem. The coping strategies are divided into three groups – stress, emergency, and crisis.

## Declaration of Competing Interest

The authors declare that they have no known competing financial interests or personal relationships which have, or could be perceived to have, influenced the work reported in this article. R.N. is deceased.
